# Two-Year Outcomes of Vagal Nerve Blocking (vBloc) for the Treatment of Obesity in the ReCharge Trial

**DOI:** 10.1007/s11695-016-2325-7

**Published:** 2016-08-10

**Authors:** Caroline M. Apovian, Sajani N. Shah, Bruce M. Wolfe, Sayeed Ikramuddin, Christopher J. Miller, Katherine S. Tweden, Charles J. Billington, Scott A. Shikora

**Affiliations:** 1 Boston University School of Medicine, 88 East Newton Street, Boston, MA 02118 USA; 2 Tufts Medical Center, Boston, 800 Washington St, Boston, MA 02111 USA; 3 Oregon Health & Science University, 3181 SW Sam Jackson Park Road, Portland, OR 97239 USA; 4 University of Minnesota, 420 Delaware St, Minneapolis, MN 55455 USA; 5 3D Communications, LLC, 8386 Six Forks Rd, Raleigh, NC 27615 USA; 6 EnteroMedics Inc., 2800 Patton Road, St. Paul, MN 55113 USA; 7 University of Minnesota, Minneapolis, Minnesota Veterans’ Administration Medical Center, One Veterans Drive, Minneapolis, MN 55417 USA; 8 Brigham and Women’s Hospital, 75 Francis Street, Boston, MA 02115 USA

**Keywords:** Obesity, Weight loss surgery, Vagal nerve blocking, Quality of life

## Abstract

**Background:**

The ReCharge Trial demonstrated that a vagal blocking device (vBloc) is a safe and effective treatment for moderate to severe obesity. This report summarizes 24-month outcomes.

**Methods:**

Participants with body mass index (BMI) 40 to 45 kg/m^2^, or 35 to 40 kg/m^2^ with at least one comorbid condition were randomized to either vBloc therapy or sham intervention for 12 months. After 12 months, participants randomized to vBloc continued open-label vBloc therapy and are the focus of this report. Weight loss, adverse events, comorbid risk factors, and quality of life (QOL) will be assessed for 5 years.

**Results:**

At 24 months, 123 (76 %) vBloc participants remained in the trial. Participants who presented at 24 months (*n* = 103) had a mean excess weight loss (EWL) of 21 % (8 % total weight loss [TWL]); 58 % of participants had ≥5 % TWL and 34 % had ≥10 % TWL. Among the subset of participants with abnormal preoperative values, significant improvements were observed in mean LDL (−16 mg/dL) and HDL cholesterol (+4 mg/dL), triglycerides (−46 mg/dL), HbA1c (−0.3 %), and systolic (−11 mmHg) and diastolic blood pressures (−10 mmHg). QOL measures were significantly improved. Heartburn/dyspepsia and implant site pain were the most frequently reported adverse events. The primary related serious adverse event rate was 4.3 %.

**Conclusions:**

vBloc therapy continues to result in medically meaningful weight loss with a favorable safety profile through 2 years.

**Trial Registration:**

https://clinicaltrials.gov/ct2/show/NCT01327976

## Introduction

Intermittent vagal blockade (vagal blocking device [vBloc] therapy) was developed as a less invasive alternative to standard bariatric surgery. It was conceived and developed based on prior reports of vagotomy as a treatment for obesity [1, 2]. Its mechanism of action is thought to involve reducing sensations of hunger. The device, the Maestro Rechargeable System, delivers low energy, high frequency, intermittent, electrical pulses to the intra-abdominal vagal trunks for a predetermined number of hours each day and is implanted using standard minimally invasive laparoscopic surgical techniques. Previous reports of randomized clinical trials on vBloc therapy have demonstrated meaningful weight loss, improvement in obesity related comorbid conditions such as type 2 diabetes mellitus (T2DM), and a low rate of serious complications [3–5].

The effect of vBloc on weight loss in the ReCharge Trial has been previously reported up to 18 months [3, 6]. The estimated mean percentage of excess weight loss (%EWL) was 26 % (10 % total weight loss [%TWL]) for vBloc and 17 % (6 % TWL) for Sham at 12 months (*p* < 0.001). From 12 to 18 months, the vBloc arm maintained its weight loss while the Sham arm regained 40 % of weight lost at 12 months, so that at 18 months the mean %EWL was 24 % (9 % TWL) for vBloc versus 10 % EWL (4 % TWL) for the Sham arm (*p* < 0.001) [6]. Sham participants crossed over to active vBloc therapy or withdrew from the study after the 18-month visit. Documentation of the longer-term durability of weight loss, improvements in comorbidities, and safety of vBloc therapy is required. This report summarizes safety and efficacy data from the vBloc arm of the ReCharge Trial at 24 months.

## Methods

### Participants

The study design and methods of the ReCharge Trial study have been described previously [3, 6]. The study participants were enrolled both in the USA (eight sites) and in Australia (two sites). BMI inclusion criteria were 35 to 40 kg/m^2^ with at least one obesity-related comorbidity (T2DM, hypertension, dyslipidemia, sleep apnea syndrome, or obesity related cardiomyopathy) or BMI of 40 to 45 kg/m^2^ with or without comorbidities. Prior to enrollment, the trial protocols were approved by the Institutional Review Boards at each site and informed consent was collected from all participants.

### Study Design

The ReCharge Trial is a double-blind, randomized controlled trial comparing vBloc therapy delivered to the intra-abdominal vagal trunks via the Maestro Rechargeable System to a sham surgical procedure with the implantation of a sham device. The primary efficacy and safety outcomes were assessed at 12 months. Following the completion of all 12 month visits, participants were unblinded to their treatment assignment. Patients randomized to the Sham arm were given the option of crossing over to receive active vBloc therapy or withdrawing from the trial. Participants randomized to the vBloc arm continued to receive open-label vBloc therapy. Study design mandates that all participants in the ReCharge Trial are to be followed for a total of 5 years following implantation with an active vBloc device.

The safety of the study was monitored by an independent Data and Safety Monitoring Board, and all serious adverse events (SAEs) were independently adjudicated for relatedness by an independent clinical events committee (CEC). The study received institutional review board (IRB) or ethics committee (EC) approval from Bellberry Limited EC, Scottsdale Clinical Research Institute Scottsdale Healthcare, Tufts Medical Center IRB, Oregon Health & Science University IRB, Mayo Clinic Rochester IRB, Stanford University Medical Center IRB, University of Minnesota IRB, Scripps IRB, and Western IRB. The study was registered on clinicaltrials.gov with the identifier NCT01327976.

### Intervention

The Maestro Rechargeable System consists of two leads placed around the anterior and posterior vagal trunks near the gastroesophageal junction using standard laparoscopic surgery and a rechargeable neuroregulator which is placed subcutaneously on the thoracic wall. The device is recharged transcutaneously [7]. Devices were programmed to 13 h of therapy per day to deliver at least 12 h of therapy daily since therapy cannot be delivered during recharging. The goal current amplitude was 6 mA. Investigators could adjust daily therapy duration and/or current amplitude based on weight loss and therapy tolerability. The average therapy delivery per day through 2 years was 11.5 ± 3.2 h at an average current amplitude of 5.9 ± 1 mA.

Monthly follow-up visits occurred between 12 and 24 months with a 2-week visit window. At each clinic follow-up visit, all participants were asked to participate in 15-min individual educational discussions on healthy food choices, exercise, and behavioral modification. Additionally, group weight management sessions were held approximately every 3 months. Of note, no new weight management material was provided in the second year of the trial; weight management advisors revisited topics at their discretion that had been discussed in the first year of the trial.

### Study Objectives

The objective of the current report was to evaluate the impact of vBloc therapy on weight loss, obesity-related comorbid conditions, quality of life, and safety at 24 months. The Sham arm is no longer a valid comparator to the vBloc group at 2 years given the appreciable rate of either cross-over to an active vBloc device or withdrawal from the trial. Weight loss was assessed as %EWL using the BMI 25 kg/m^2^ method and %TWL. At every study visit, adverse events were collected using standard case report forms to capture the event type, investigator-attributed relatedness, seriousness, and severity.

Systolic and diastolic blood pressures were measured at baseline and at every visit. Blood pressure (in triplicate), laboratory parameters, and waist circumference were assessed at baseline and at yearly visits. The laboratory parameters of focus in this report were those known to be improved with significant weight loss: total cholesterol, high density lipoprotein (HDL) cholesterol, low density lipoprotein (LDL) cholesterol, triglycerides, fasting plasma glucose (FPG), and hemoglobin A1c (HbA1c). The ReCharge Trial did not require that patients have elevated levels of these parameters to be eligible for enrollment, and many patients were well controlled on medications throughout the trial. However, a significant number of participants presented with abnormal metabolic and cardiovascular parameters, therefore, in addition to assessing change in these parameters among all participants, we also examined the change among participants with abnormal (i.e., elevated or low) levels of these parameters at baseline.

Quality of life (QOL) was assessed at baseline and every 6 months using the Impact of Weight on Quality of Life-Lite (IWQOL-Lite), a validated QOL instrument with 31 questions that measures five domains: physical function, self-esteem, sexual life, public distress, and work [8]. Scores range from 0 to 100, with higher scores indicating better QOL. Changes in eating were evaluated using the Three-Factor Eating Questionnaire (TFEQ) at baseline and every 6 months. The TFEQ is a validated, self-report questionnaire that is used to measure the psychological constructs of eating on three subscales: cognitive restraint (0–21 scale), disinhibition (0–16 scale), and hunger (0–14 scale) [9].

### Pre-Diabetes and Metabolic Syndrome Assessment

Exploratory assessments were done to determine if participants who presented with either pre-diabetes or metabolic syndrome at baseline still had the syndrome at 12 and 24 months. Pre-diabetes was defined according to the American Diabetes Association (ADA) diagnostic criteria for elevated FPG (≥100 to 125 mg/dL) and/or elevated HbA1c (≥5.7 and <6.5 %) for patients without diabetes and not on medications for endocrine disorders [10].

The metabolic syndrome definition used was taken from the National Cholesterol Education Program (NCEP) with the presence of three of the following conditions: elevated waist circumference (men >102 cm, women >88 cm), elevated triglycerides (≥150 mg/dL), low HDL (men <40 mg/dL, women <50 mg/dL), elevated blood pressure (≥130/85 mmHg or use of hypertension medications), and elevated FPG (≥110 mg/dL) [11].

### Statistical Analysis

No statistical analysis plan was pre-specified for evaluation of changes after the 12-month primary assessment. For this report, the statistical significance of changes in weight and other continuous parameters in the vBloc group were evaluated using paired *t* tests comparing participants’ baseline values to their follow-up values. No statistical hypothesis tests were used for categorical parameters. All analyses are reported as complete case analyses without imputation or adjustment for multiple comparisons. *P* values less than 0.05 were considered statistically significant. All statistical analyses were performed using SAS version 9.3.

## Results

### Baseline Characteristics and Participant Disposition

The baseline characteristics of the ReCharge Trial patient population have been summarized previously [3]. Of the 162 participants randomized to the vBloc group, 87 % were female, the mean age was 47 years, the mean BMI was 41 kg/m^2^_,_ 39 % had hypertension, 56 % had dyslipidemia, and 20 % had obstructive sleep apnea. Nine participants (6 %) had T2DM.

Seventy-six percent of the randomized vBloc participants (*n* = 123) remained in the trial at 24 months. The reasons for withdrawals in the vBloc group were as follows: 23 (14.2 %) subject decisions, 9 (5.6 %) for an adverse event, 2 lost to follow-up (1.2 %), and the other 5 due to intra-operative exclusions as a result of which the vBloc device was not implanted, which has been described previously [3]. The withdrawals for an adverse event were due to pain at the neuroregulator site in five cases, the need for MRI in two cases, heartburn in one case, and abdominal pain in another. As previously reported, eight participants required nine revisions in the first year of the study [3]. There were four additional revisions between 12 and 24 months: two due to the adverse event of pain at the neuroregulator site, one due to twisted leads caused by the participant chronically rotating the neuroregulator in the subcutaneous pocket (“Twiddler’s syndrome”) where both leads and the neuroregulator were replaced, and one due to the inability to consistently recharge the neuroregulator which a neuroregulator replacement remedied. All revisions were uncomplicated, and the patients were released on the day of or day following the procedure.

### Weight Loss

The mean EWL among vBloc participants who presented for the 24-month visit was 21 % (95 % CI 16 to 26 %); the mean percent TWL was 8 % (95 % CI 6 to 10 %). Twenty participants did not present for the visit but remained in the study. The percentage of participants who achieved various TWL thresholds from at least 5 % to at least 15 % TWL were similar at 12 and 24 months (Table 1). Only 24 participants of the 77 randomized to the Sham arm remained in the trial who had not yet crossed over to active vBloc therapy. However, the mean weight loss among these individuals was only 4 % EWL (1 % TWL).

**Table 1:** Percentage of total body weight loss thresholds

%TWL achieved	vBloc at 12 months (*N* = 147)	vBloc at 18 months (*N* = 117)	vBloc at 24 months (*N* = 103)
≥5.0 %	98 (67 %)	80 (68 %)	60 (58 %)
≥7.5 %	82 (56 %)	64 (55 %)	46 (45 %)
≥10.0 %	57 (39 %)	46 (39 %)	35 (34 %)
≥12.5 %	47 (32 %)	37 (32 %)	28 (27 %)
≥15.0 %	33 (22 %)	30 (26 %)	22 (21 %)

Complete case analysis

### Improvements in Cardiovascular, Anthropometric, and Metabolic Parameters

Mean screening values for cardiovascular and anthropometric risk factors with average changes at yearly follow-up visits for all vBloc participants who presented for the visit and for the subset of participants with abnormal screening values are provided in Table 2. Overall, the improvements from baseline in systolic and diastolic blood pressure and waist circumference were significant; those with elevated blood pressure at baseline had two to three times greater improvements, which were also statistically significant.

**Table 2:** Changes in cardiovascular and anthropometric obesity risk factors at 12 and 24 months

Risk factor	Time point	All vBloc participants	vBloc participants with abnormal baseline values
Systolic blood pressure (mmHg)	Screening	128 ± 13 (162)	142 ± 10 (40)
	12 month change	-*6* [−*8*, −*3*] (*147*)	-*15* [−*20*, −*11*] (*36*)
	24 month change	-*6* [−*8*, −*3*] (*102*)	-*11* [−*17*, −*6*] (*29*)
Diastolic blood pressure (mmHg)	Screening	81 ± 9 (162)	89 ± 8 (40)
	12 month change	-*3* [−*4*, −*1*] (*147*)	-*10* [−*12*, −*7*] (*36*)
	24 month change	-*3* [−*5*, −*1*] (*102*)	-*10* [−*14*, −*6*] (*29*)
Waist circumference (cm)	Screening	121 ± 12 (161)	123 ± 11 (151)
	12 month change	-*10* [−*12*, −*9*] (*143*)	-*11* [−*12*, −*9*] (*134*)
	24 month change	-*8* [−*11*, −*6*] (*102*)	-*10* [−*12*, −*8*] (*95*)

Data are presented as screening value means ± SD (*N*), or as mean change from screening [95 % CI] (*N*). Italics font indicates that the change is significant at the *P* < 0.05 level. Abnormal screening values for cardiovascular and anthropometric risk factors were as follows, respectively: systolic blood pressure ≥ 140 mmHg or diastolic blood pressure≥90 mmHg; waist circumference >102 cm for men, and >88 cm for women. Data at 12 and 24 months are from participants who presented at visit and from which the appropriate data were collected

Mean screening values and 12- and 24-month mean changes for metabolic obesity risk factors are shown for all vBloc participants who presented for the visit as well as those with abnormal screening values in Table 3. Among all vBloc participants, 24-month improvements from baseline were statistically significant for LDL cholesterol, HDL cholesterol, triglycerides, and HbA1c. Improvements were greater for all parameters among participants whose values were abnormal at baseline. Fasting plasma glucose was the only metabolic parameter that was not significantly impacted at 24 months. Among the subset of participants who met the criteria for metabolic syndrome at baseline, 50 % remitted from that diagnosis at 12 months and 47 % by 24 months (Table 4). Similarly, among the participants who were pre-diabetic at screening, 57 % had normal glucose profiles at 12 months and 50 % had normal values at 24 months (Table 5).

**Table 3:** Changes in metabolic obesity risk factors at 12 and 24 months

Risk factor	Time point	All vBloc participants	vBloc participants with abnormal baseline values
Total cholesterol (mg/dL)	Screening	204 ± 36 (150)	235 ± 25 (63)
	12 month change	-*9* [−*14*, −*4*] (*145*)	-*20* [−*28*, −*12*] (*62*)
	24 month change	-5 [−10, 1] (97)	-*17* [−*25*, −*9*] (*43*)
LDL cholesterol (mg/dL)	Screening	122 ± 32 (150)	151 ± 21 (63)
	12 month change	-*5* [−*10*, −*1*] (*145*)	-*15* [−*22*, −*7*] (*62*)
	24 month change	-*5* [−*10*, −*1*] (*97*)	-*16* [−*23*, −*9*] (*43*)
HDL cholesterol (mg/dL)	Screening	54 ± 14 (150)	36 ± 3 (19)
	12 month change	1 [−1, 3] (145)	*5* [*2*, *7*] (*19*)
	24 month change	*3* [*2*, *5*] (*97*)	*4* [*0*, *8*] (*12*)
Triglycerides (mg/dL)	Screening	139 ± 61 (150)	209 ± 42 (52)
	12 month change	-*22* [−*31*, −*12*] (*145*)	-*48* [−*68*, −*29*] (*51*)
	24 month change	-*14* [−*24*, −*3*] (*97*)	-*46* [−*63*, −*28*] (*34*)
Fasting plasma glucose (mg/dL)	Screening	96 ± 17 (137)	129 ± 23 (15)
	12 month change	-2 [ −4, 1] (130)	-*15* [−*29*, −*1*] (*14*)
	24 month change	1 [ −3, 5] (81)	-15 [−34, 4] (10)
Hemoglobin A1c (%)	Screening	5.7 ± 0.6 (149)	5.9 ± 0.2 (53)
	12 month change	-*0*.*3* [ −*0*.*4*, −*0*.*3*] (*144*)	-*0*.*4* [−*0*.*5*, −*0*.*4*] (*52*)
	24 month change	-*0*.*3* [ −*0*.*4*, −*0*.*2*] (*95*)	-*0*.*3* [−*0*.*5*, −*0*.*2*] (*39*)

Data are presented as screening value means ± SD (*N*), or as mean change from screening [95 % CI] (*N*). Italics font indicates that the change is significant at the *P* < 0.05 level. Abnormal screening values for metabolic risk factors were as follows, respectively: total cholesterol >200 mg/dL; LDL cholesterol >130 mg/dL; HDL cholesterol <40 mg/dL; triglycerides >150 mg/dL; fasting plasma glucose ≥110 mg/dL, hemoglobin A1c ≥5.7 %. Data at 12 and 24 months are from participants who presented at visit and from which the appropriate data were collected

**Table 4:** Changes in metabolic syndrome at 12 and 24 months by the National Cholesterol Education Program (Adult Treatment Panel III) definition

Metabolic syndrome status	vBloc at 12 months *N* = 130	vBloc at 24 months *N* = 81
Normal status at baseline	*N* = 74	*N* = 47
Normal at follow-up	64 (86 %)	40 (85 %)
Developed metabolic syndrome	10 (14 %)	7 (15 %)
Metabolic syndrome at baseline	*N* = 56	*N* = 34
Improved to normal at follow-up	28 (50 %)	16 (47 %)
Retained metabolic syndrome	28 (50 %)	18 (53 %)

Metabolic syndrome defined as the presence of three or more of the following risk determinants: (1) increased waist circumference (>102 cm for men, >88 cm for women); (2) elevated triglycerides (≥150 mg/dL); (3) low HDL cholesterol (<40 mg/dL in men, <50 mg/dL in women); (4) hypertension (≥130/85 mmHg); and (5) impaired fasting glucose (≥110 mg/dL)

**Table 5:** Changes in pre-diabetic status at 12 and 24 months by the American Diabetes Association definition

Pre-diabetic status	vBloc at 12 months *N* = 109	vBloc at 24 months *N* = 71
Normal* at baseline	*N* = 55	*N* = 37
Normal at follow-up	48 (87 %)	29 (78 %)
Developed pre-diabetes	7 (13 %)	8 (22 %)
Pre-diabetes at baseline	*N* = 54	N = 34
Improved to normal at follow-up	31 (57 %)	17 (50 %)
Retained pre-diabetes	23 (43 %)	17 (50 %)

*Normal status defined as having both fasting plasma glucose (FPG) <100 mg/dL and HbA1c <5.7 %. Pre-diabetes defined as FPG ≥100 mg/dL or HbA1c ≥5.7 %. Excludes patients with missing screening or 24-month lab values, patients diagnosed as diabetic at baseline, and patients who were on diabetic medications at screening or through the 24-month visit

### Quality of Life and Food Intake-Behavior

Significant improvements from baseline were observed in the IWQOL-Lite questionnaire at both 12 and 24 months among participants who received vBloc therapy (Table 6). From a mean score of 57 (on a 0 to 100 scale, where higher scores indicate greater quality of life), the mean improvement from screening was 20 points at both yearly visits.

**Table 6:** Three-Factor Eating Questionnaire (TFEQ) and Impact of Weight on Quality of Life-Lite (IWQOL-Lite) screening values and 12- and 24-month change

Quality of life parameter	Time point	Mean ± SD (N) or mean change [95 % CI] (*N*)
IWQOL-Lite (0–100 scale)	Screening	57 ± 17 (157)
	12 month change	*20* [*17*, *23*] (*142*)
	24 month change	*20* [*17*, *24*] (*100*)
TFEQ: hunger (0–14 scale)	Screening	8.0 ± 3.3 (160)
	12 month change	-*4*.*1* [−*4*.*8*, −*3*.*5*] (*145*)
	24 month change	-*4*.*1* [−*5*.*0*, −*3*.*3*] (*100*)
TFEQ: disinhibition (0–16 scale)	Screening	10.3 ± 3.3 (160)
	12 month change	-*3*.*3* [−*4*.*0*, −*2*.*7*] (*145*)
	24 month change	-*3*.*0* [−*3*.*8*, −*2*.*3*] (*100*)
TFEQ: cognitive restraint (0–21 scale)	Screening	9.5 ± 4.4 (160)
	12 month change	*5*.*8* [*5*.*1*, *6*.*6*] (*145*)
	24 month change	*6*.*0* [*5*.*1*, *6*.*9*] (*100*)

Italics font indicates that the change is significant at the *P* < 0.05 level. Data at 12 and 24 months are from participants who presented at visit and from which the appropriate data were collected

As shown in Table 6, each of the three factors on the TFEQ results improved significantly from baseline. The hunger factor, of greatest interest since decreasing hunger and earlier satiety are considered to be the mechanism of action of vBloc therapy [7], was significantly decreased from a mean of 8 points (on a 0–14 scale, where higher scores indicate experiencing more sensations of hunger) at screening to 4 points at 12 months, which was sustained at 24 months. The disinhibition factor, which measures the ability to control emotional or social eating, and cognitive restraint, which assesses the ability to avoid weight-gaining behaviors by limiting consumption, was also significantly improved.

### Safety

The related adverse event profile of the vBloc device cumulatively through 24 months was similar to that reported at 12 months (Table 7) [3]. The most frequently reported related adverse events were heartburn and dyspepsia, neuroregulator site pain, other pain, abdominal pain, incision pain, nausea, eructation/belching, and dysphagia. Ninety-four percent of all adverse events were reported as mild or moderate in severity, and 83 % of events had resolved by 24 months.

**Table 7:** Adverse events related to device, procedure, or therapy through 24 months

AE type	*N* participants (%)	*N* events	Resolved (%)	Mild/moderate severity (%)
Pain, neuroregulator site	61 (38)	78	82	96
Other	42 (26)	52	77	96
Pain, other	41 (25)	50	68	96
Heartburn/dyspepsia	42 (26)	47	60	100
Pain, abdominal	23 (14)	31	84	100
Nausea	13 (8)	18	89	89
Chest pain	15 (9)	16	69	94
Dysphagia	14 (9	14	79	100
Eructation/belching	14 (9)	14	71	100
Incision pain	12 (7)	14	100	100
Bloating, abdominal	7 (4)	9	78	100
Constipation	8 (5)	8	88	88
Cramps, abdominal	8 (5)	8	50	100
Wound redness or irritation	8 (5)	8	100	100
Emesis/vomiting	6 (4)	8	100	88

Only adverse events attributed by the investigator to the device, procedure, or therapy that occurred in at least 5 % of vBloc group participants are displayed

All primary endpoint-related serious adverse events have been previously reported through 18 months, so the rate remained constant at 4.3 % [3, 6]. Three serious adverse events of infection, confusion with hallucinations, and brain tumor were reported and adjudicated by the independent clinical events committee to be unrelated to vBloc therapy.

## Discussion

Intermittent vagal blockade using the vBloc device for 24 months in the ReCharge Trial demonstrated sustained weight loss, significant improvements in obesity related cardiovascular and metabolic parameters, and a low rate of significant adverse events. Vagal block treated participants achieved 21 % EWL (8 % TWL) at 24 months, similar to the weight loss at 12 and 18 months [3, 6]. In the vBloc-treated participants, significant improvements from baseline in blood pressure, lipids, and glycemic control were observed at 24 months; and, among participants beginning with abnormal values, the improvements were even more substantial. The prevalence of pre-diabetes and metabolic syndrome was halved among those participants who had those conditions at baseline. Most adverse events were reported as mild or moderate in severity, and there were no serious long-term consequences, such as nutritional deficiencies, which have been reported for conventional bariatric surgery.

Quality of life and eating behaviors of vagal blocking therapy participants were significantly improved. Measures from the IWQOL-Lite suggest that the weight loss achieved by participants treated with vBloc led to clinically meaningful improvements in the impact of weight and obesity on the quality of their lives. As had been hypothesized, results from the TFEQ suggest that a substantial reduction in hunger (i.e., feelings of satiety) were achieved and sustained, with identical reductions in the average hunger factor of the TFEQ at both 12 and 24 months. Likewise, measures of disinhibition and cognitive restraint in the TFEQ improved, suggesting that vagal block treated participants felt they had better control over emotional eating and weight-gaining behaviors.

vBloc therapy results in less weight loss than has been reported with the conventional procedures sleeve gastrectomy and gastric bypass. However, in considering the comparative benefit/risk profile, the weight loss achieved with vBloc led to improvements in comorbidities (notably pre-diabetes and metabolic syndrome), quality of life, and control over hunger with fewer risks than the conventional bariatric procedures. For example, complications observed in the STAMPEDE Trial of gastric bypass and sleeve gastrectomy compared to medical therapy for the treatment of diabetes through 3 years included intra-abdominal bleeding (and subsequent need for transfusion), gastrointestinal leak, bowel obstruction, dehydration requiring intravenous treatment, stricture, ulcers, and ketoacidosis [12, 13]. Weight loss with vBloc was shown to be similar to that reported for laparoscopic adjustable gastric banding (LAGB) in a 2-year report comparing bypass, sleeve, and LAGB where the completed case evaluation showed 6 ± 8.2 % TWL with LAGB compared to 8 ± 9.5 % TWL with vBloc [14].

The limitations of the present report include missing data and the lack of a blinded control group. The ReCharge Trial has been unblinded, and participants are now receiving open-label vBloc therapy, so the study is subject to the limitations inherent in an uncontrolled design with more limited follow-up. It is reassuring that the magnitude of the improvements from baseline at 24 months in weight loss, cardiovascular risk and metabolic parameters, and patient-reported measures are similar to 12 months, when the blind and sham control were still in effect.

## Conclusions

Intermittent vagal blockade with vBloc therapy produces medically meaningful weight loss through 2 years, with a favorable safety profile and sustained improvements in obesity-related cardiovascular risk factors, healthy eating behaviors, and quality of life.
